# On balance: lifestyle, mental health and wellbeing

**DOI:** 10.1057/palcomms.2016.75

**Published:** 2016-10-18

**Authors:** Ali Haggett

**Affiliations:** 1 Centre for Medical History, University of Exeter, Exeter, UK

## Abstract

Given the supremacy of the biomedical model in defining our understanding and treatment of a wide range of physcial and psychological disorders, it is perhaps curious that simultaneously, scientists, clinicians, governments and patients routinely employ the concepts of “lifestyle” and “balance” to try to explain the causes of bodily disease and psychological disorder. Concurrently, the health advantages that are assumed to be inherent in a “balanced life” have been exploited by a rapidly expanding consumer market in “wellbeing”—by companies and individuals promoting food supplements, “wearable fitness”, diet trends and the self-help material. Exploring the tension between the biomedical doctrine and the parallel preoccupation with balance and lifestyle has provided the impetus for this special issue. Emerging originally from papers presented at an interdisciplinary conference held at the University of Exeter in June 2015, and augmented by two further comment pieces, the collection of articles aims to explore the ways in which changing notions of “balance” have been used to understand the causes of mental illness; to rationalise new approaches to its treatment; and to validate advice relating to balance in work and family life.

The concept of “balance” in health and medicine has a long and noteworthy history. Even those with no more than a cursory knowledge of medical history will probably be familiar with the theories of Hippocrates (c. 460 BC–c. 375 BC) and Galen (c. 129 AD–c. 210 AD), and the practice of humoral medicine, which was dominant in the West from Antiquity up until the developments in microbiology during the eighteenth and nineteenth centuries.^1^ Central to humoral medicine was the notion of balance, or “equilibrium”, whereby health required the correct balance of the four humours: black bile, yellow bile, blood and phlegm. Contrastingly, it was “imbalance” of these humours that was thought to result in disease. Each humour was also innately associated with the seasons and the environment and thought to have the characteristics of heat, cold, dry or wet. Regimens (or treatments) were designed to restore balance, by purging, bloodletting, inducing vomiting or the administering of enemas. “Madness” too, was understood in humoral terms: “mania” caused by an excess of choler (yellow bile), which caused irascible or excited behaviour, while an excess of black bile was thought to lead to melancholia, causing mental and physical symptoms that we might now recognise as depression (Porter, 1999, Chapter 3; Radden, 2000, Chapter 2).

During this period, psyche and soma were not regarded as distinct entities, and it was accepted that the emotions profoundly influenced bodily functions. Indeed, according to Galen the term “psychogenesis” indicated “passion-produced” disease (Furst, 2012: 20–21). Lifestyle, or “regimen”, was also central to prevention of disease. The balance (or regulation) of diet, exercise, rest and sleep, excretion and retention of fluids, airs, and passions of the soul—and knowledge of one’s own constitution—were key to health. In contrast, ill-health was seen as a consequence of poor self-control and the cultivation of bad habits (Spary, 2011: 86, 87.)

During the eighteenth and nineteenth centuries, the nature and philosophy of medicine changed significantly as new discoveries were made about morbid anatomy and disease. The change of medical focus from lifestyle, regimens and humoral medicine presented “a powerful model for understanding illness that still survives, and which became known as biomedicine” (Hardy, 2001: 4). Indeed, throughout the twentieth century, and particularly during the decades following the Second World War, significant achievements were made in the fields of surgery, bacteriology and pharmacology, consolidating gains made over the previous two hundred years and reinforcing the increased confidence in curative, interventionist medicine (Haggett, 2015: 8). Since the 1950s, the treatment of what we now understand to be mental illness has been largely dominated by biological psychiatry and the development of new drugs to treat severe psychological disorders and mild-to-moderate anxiety and depression. The doctrine underlying the use of these drugs suggests that mental disorders are caused by a “chemical imbalance” in the brain and that psychotropic medication targets and reverses the imbalance. Despite criticism of this concept among a vocal minority (for example, Healy, 2004, 2012; Moncrieff, 2008,^2013^; Bentall, 2009; Kinderman, 2014), the biochemical model of mental illness continues to dominate our understanding and treatment of a wide range of psychological disorders.

Given the supremacy of the biomedical model, it is perhaps curious that simultaneously, scientists, clinicians, governments and patients routinely employ the concepts of “lifestyle” and “balance” to try to explain the causes of bodily disease and psychological disorder. Concurrently, the health advantages that are assumed to be inherent in a “balanced life” have been exploited by a rapidly expanding consumer market in “wellbeing”—by companies and individuals promoting food supplements, “wearable fitness”, diet trends and the self-help material. Exploring the tension between the biomedical doctrine and the parallel preoccupation with balance and lifestyle has provided the impetus for this special issue (article collection) of *Palgrave Communications*. Emerging originally from papers presented at an interdisciplinary conference held at the University of Exeter in June 2015, and augmented by two further comment pieces, the collection aims to explore the ways in which changing notions of “balance” have been used to understand the causes of mental illness; to rationalise new approaches to its treatment; and to validate advice relating to balance in work and family life. Drawing on a range of different approaches and methodologies, the articles are authored by scholars from diverse backgrounds: anthropology, psychology and the history of medicine.

Collectively, the contributions focus (historically and contemporarily) on debates about the causes of poor psychological health; how we have attempted to resolve the problem; and what we might do in the future. Many of the broader anxieties about health, wellbeing and lifestyle that are explored in the articles and comment pieces about modern life are not new, but have emerged at regular junctures in our recent history. A number of authors, for example, highlight the importance of viewing illness in social context. As Kinderman (2016) argues in his comment piece, the social determinants of mental ill-health should be more prominently emphasised. Poverty, unemployment, childhood trauma and dysfunctional relationships have indeed been proven in epidemiological studies to be direct causes of mental illness. These concerns are reminiscent of those put forward much earlier, during the early and mid-twentieth century, by proponents of the social medicine movement who were critical of rising consumerism, the breakdown of traditional values and kinship ties, and who were keen to reduce the burden of sickness by pressing for social improvements (for example: Taylor, 1938; Halliday, 1948; Engel, 1977). As Smith (2016) illustrates in his article on the post-war community mental health movement in the United States, these ideas were also salient during the Kennedy and Johnson administrations, where social welfare reforms not only moved to tackle poverty, but also mental illness—the cause of which was seen to be in “harsh environmental conditions”.

Smith’s (2016) paper explores the wider issue of how societies have attempted to “balance” individualism with the needs of the broader population. As he rightly notes, we often think of balance in terms of “individual” responsibility, whether it be balancing humours, a balanced diet or work-life balance. However, the concept is perhaps also useful for understanding how societies have approached matters of public health—whereby certain individual freedoms are curtailed in order to achieve the broader goal of improved health for the entire population. American post-war social psychiatry indeed shifted attention from the individual (and the immediate family environment) to communities and the broader social environment. Using a study of Community Mental Health Centres, Smith explores the shift from asylum to community care, where the focus on prevention prompted a shift in psychiatric practice.

Davies (2016) also draws on tensions between the individual and the group in his study of current approaches to mental health disorders in the workplace. Through narrative analysis of resources produced from a range of organisations that advise on workplace distress management, Davies shows convincingly that current neoliberal working practices locate the causes of employee distress and despondency in the individual and their “micro-working surroundings”, while underplaying broader political and social structural pressures that impinge on working life. Thus, workplace dissatisfaction and disengagement are increasingly ignored and reframed as mental ill-health, rooted in psycho-biological factors and treated with therapeutic interventions.

Working life also provides the foundation to Cooper’s (2016) historical study of post-war medical debates about women and work during the 1950s. This revisionist account examines the work of Medical Women’s International Association and the British Medical Women’s Federation, illustrating that, in some clinical quarters, paid employment was viewed as an important solution to female dissatisfaction. Challenging previous histories that have tended to assume medical opinion secured and reinforced the notion that a woman’s place was in the home, Cooper shows that the view of some clinicians was that paid work provided stimulation and fulfilment, which was essential to a woman’s wellbeing. This article also draws on tensions highlighted by Smith and Davies about the balance between the individual and the group, since many of the anxieties about women’s participation in paid work were couched in discourse that suggested women who worked risked the health and stability of their families—and should therefore be implicated in broader familial and social breakdown. Contemporary commentators, as Cooper illustrates, argued that the answer was ultimately about balance: intrapersonal balance between different sources of fulfilment and identity, and equilibrium between the demands of the community and the needs of the individual.

In Nathoo’s (2016) article, the focus shifts towards ways of “rebalancing” the self. This historical paper explores the emergence of relaxation as a tool for balancing overactive bodies, minds and emotions, from the interwar period to the 1970s in Britain. Nathoo demonstrates how a set of broader anxieties about “modern” and increasingly stressful ways of living provided the perfect context within which theories about the importance of relaxation could flourish. Particularly influential in the field of obstetrics, the practice of relaxation both established and transformed the field of ante-natal care. Also of note was its use in the prevention of heart disease, which was reconceptualised during the 1960s as preventable (if individuals chose to moderate their lifestyles and consumption habits). Reflecting observations made in a number of the other papers, Nathoo notes that the field of social medicine, by the 1960s had begun to identify the cause of ill health in the individual and his or her lifestyle, as opposed to seeing it as rooted in social structures. This study, which focuses on the origins and activities of Britain’s first relaxation charity, Relaxation for Living, illustrates neatly how the practice evolved through the period from “a disparate set of activities and meanings” to a “technical, therapeutic skill that had to be taught, learnt and practiced”. Crucially, the paper sets these developments within the context of a health service increasingly modelled on interventionist medicine, focused on treating “disease”. And as Nathoo pertinently notes, the appeal of cost-effective, nonpharmaceutical methods of coping with stressful lives continues to be as relevant today as it was almost a century ago.

Implicit in a number of the contributions to this special issue is the notion that the current biomedical model of mental illness tends to foreground treatment and underplay prevention. As Smith’s article demonstrates, increasingly the emergence of psycho-pharmaceutical treatments for mental illness reinforced an interventionist model of psychiatric disorder, while undermining the importance of environmental approaches to wellbeing. Concurrently, biological psychiatry has helped to reorient the gaze from populations to individuals. Many would agree with Smith and Kinderman’s analyses, that the failure to focus on social justice has hampered attempts to improve the mental health of populations. Kinderman notes that people are routinely offered powerful drugs, but struggle to access evidence-based psychological therapies or practical help to solve real-world issues such as debt, unemployment, housing problems and domestic violence. Although psychiatry neuropsychiatry and psychopharmacology will doubtless continue to provide important contributions to care of those with mental health problems, a narrow biological approach entirely underplays the public health dimension (Bentall, 2016).

Psychology offers new and innovative approaches to supporting those with mental health problems, and for preventing relapse. Tim Tomas, for example, illustrates in his comment piece on Second Wave Positive Psychology, how scholars have begun to build on the work of individuals such as Martin Seligman, whose work showed that concepts of happiness and flourishing were largely absent from mainstream psychology (Lomas, 2016). Positive psychology, originally coined by Abraham Maslow in his book *Motivation and Personality* (1954). But developed later by Seligman and others, emphasised growth and achievement, as opposed to pathology (Seligman, 1991). However, as Lomas (2016) demonstrates, critics were cautious that the emphasis on positivity led to a cultural expectation that “one *should* be upbeat”—an expectation that could also have deleterious consequences. “Second wave” positive psychology offers a more nuanced approach, which recognises that “flourishing” in fact involves a complex balance—subtle interplay—between positive and negative phenomena. Kinderman too, points out that we must acknowledge complexity and nuance when we observe individual reactions to challenging social circumstances. Individuals react differently to traumatic circumstances, based upon a mosaic of interwoven factors, for example: the level of social support available to them and learned responses which shape their view of the world. As the renowned American psychiatrist Menninger (1967: 42) noted in his book *The Vital Balance: The Life Process in Mental Health and Illness*, first published in 1963, “Illness is in part what the world has done to a victim, but in a larger part it is what the victim has done with his world and with himself”.

What is evident from these contributions is that the concept of “balance” remains at the core of all debates about mental health, whether we are talking about chemical imbalance, work-life balance or cognitive and mindful approaches to human behaviour. While historians are uniquely placed to add important context, the importance of combining insights from several disciplines is that we are able to begin to redefine problems and reach solutions through new understandings. Although not comprehensive in their coverage of topics related to mental health, the articles in this collection prompt us to think about new ways of conceptualising and measuring what is “balanced” in life and in health—and perhaps also to question the ways in which balance is somehow taken to be inherently desirable, or essential. The themes and methods provide a standpoint from which we hope scholarship might develop and extend, adding to our knowledge and debates about life, mental health and wellbeing.

## Footnotes

1 In the Easstern tradition of Chinese medicine also, “balance” is central to a set of interconnected systems, governed by “Yin and Yang”.
